# Supplementary material for: Oocyte Quality Assessment in Breast Cancer: Implications for Fertility Preservation
Source: Cancers (Basel). 2022 Nov 21;14(22):5718. doi: 10.3390/cancers14225718 (PMC9688559; doi:10.3390/cancers14225718)
Supplement: Supplementary file 1 [file cancers-14-05718-s001.zip › cancers-2036255-supplementary.pdf]

Supplementary

Oocytes quality assessment in breast cancer: implications for fertility preservation

Cristina Fabiani, Antonella Guarin, Caterina Meneghini, Emanuele Licata, Gemma Paciotti, Donatella Miriello, Michele Carlo Schiavi, Vincenzo Spina, Roberta Corno, Mariagrazia Gallo and Rocco Rago

Table S1. Variables impact on oocyte quality: uni and multivariable logistic regression according to the presence of dysmorphic oocytes in the control group.

Characteristic	Patients at risk	N° of events	Univariable		Multivariable *	
			**Odds Ratio (95% CI)**	**p value**	**Odds Ratio (95% CI)**	**p value**
Age						
Continuos	189	61	0.96 (0.88-1.05)	0.388	0.93 (0.82-1.05)	0.252
≤ 34 years	96	35	1 (Ref)		NI	-
>34 years	93	26	0.68 (0.37-1.25)	0.212		
BMI	165	56	1.03 (0.95-1.13)	0.467	1.01 (0.91-1.12)	0.814
AMH	157	50	1.02 (0.88-1.18)	0.781	1.01 (0.86-1.19)	0.859
Duration of stimulation	187	59	1.29 (1.06-1.59)	0.013	1.38 (1.06-1.79)	**0.017**
E2 level at triggering day	188	61	0.99 (0-99-1.01)	0.034	0.99 (0-99-1.01)	**0.033**
Total FSH cumulative dose	187	61	0.99 (0-99-1.01)	0.784	0.99 (0-99-1.01)	0.646

Bold font highlights statistically significant difference. CI: Confidence Interval. NI: Not included. BMI: Body Mass Index: AMH: Anti-Mullerian Hormone. E_2: Estradiol. FSH: Follicle Stimulating Hormone. * 135 patients at risk and 43 events. The variable age was included in the multivariable analysis as continuous variable; similar results were obtained using categorized age (data not shown).

Table S2. Variables impact on oocyte quality: uni and multivariable linear regression according to the number of dysmorphic oocytes.

Characteristic	Patients at risk	Univariable analysis			Multivariable analysis		
		**Coefficent (SE)**	**t**	**p value**	**Coefficent (SE)**	**t**	**p value**
Breast cancer	294	0.94 (0.21)	4.47	< 0.0001	1.15 (0.29)	4.02	< 0.0001
Age	294	-0.08 (0.03)	-2.70	0.007	-0.05 (0.03)	-1.57	0.117
BMI	270	0.01 (0.03)	0.36	0.722	0.00 (0.03)	-0.03	0.978
AMH	248	0.10 (0.05)	2.15	0.033	0.08 (0.05)	1.58	0.116
Duration of stimulation	292	-0.05 (0.05)	-0.90	0.371	0.01 (0.06)	0.20	0.845
E2 level at triggering day	292	0 (0)	0.93	0.354	0 (0)	2.13	0.034
Total FSH cumulative dose	292	0 (0)	1.15	0.250	0 (0)	-0.24	0.808

Bold font highlights statistically significant difference. SE: Standard Error. BMI: Body Mass Index. AMH: Anti-Mullerian Hormone. E2: Estradiol. FSH: Follicle Stimulating Hormone.

Table S3. Variables impact on oocyte quality: uni and multivariable linear regression according to the percentage of dysmorphic oocytes respect to the total number of retrieved oocytes per patient.

Characteristic	Patients at risk	Univariable analysis			Multivariable analysis		
		Coefficent (SE)	t	p value	Coefficent (SE)	t	p value
Breast cancer	294	4.37 (1.79)	2.44	0.015	5.66 (2.22)	2.55	**0.011**
Age	294	-0.53 (0.25)	-2.11	0.035	-0.32 (0.26)	-1.23	0.221
BMI	270	0.25 (0.26)	0.96	0.336	0.11 (0.23)	0.46	0.646
AMH	248	-0.05 (0.36)	-0.13	0.900	-0.18 (0.37)	-0.48	0.633
Duration of stimulation	292	0.43 (0.44)	0.97	0.335	-0.06 (0.48)	-0.13	0.896
E2 level at triggering day	292	0 (0)	0.12	0.904	0 (0)	1.22	0.225
Total FSH cumulative dose	292	0 (0)	1.54	0.124	0 (0)	0.54	0.588

Bold font highlights statistically significant difference. SE: Standard Error. BMI: Body Mass Index. AMH: Anti-Mullerian Hormone. E2: Estradiol. FSH: Follicle Stimulating Hormone.
